# *Withania somnifera* (L.) Dunal ameliorates neurodegeneration and cognitive impairments associated with systemic inflammation

**DOI:** 10.1186/s12906-019-2635-0

**Published:** 2019-08-15

**Authors:** Muskan Gupta, Gurcharan Kaur

**Affiliations:** Department of Biotechnology, Medical Biotechnology Laboratory, Guru Nanak Dev University, Amritsar, Amritsar, Punjab 143005 India

**Keywords:** Ashwagandha, Lipopolysaccharide, Microglia, Neuroinflammation, Neuroprotection, Synaptic plasticity

## Abstract

**Background:**

Systemic inflammation driven neuroinflammation is an event which correlates with pathogenesis of several neurodegenerative diseases. Therefore, targeting peripheral and central inflammation simultaneously could be a promising approach for the management of these diseases. Nowadays, herbal medicines are emerging as potent therapeutics against various brain pathologies. Therefore, in this contemporary study, the neuroprotective activity of Ashwagandha (*Withania somnifera*) was elucidated against the inflammation associated neurodegeneration and cognitive impairments induced by systemic LPS administration using in vivo rat model system.

**Methods:**

To achieve this aim, young adult wistar strain male albino rats were randomized into four groups: (i) Control, (ii) LPS alone, (iii) LPS + ASH-WEX, (iv) ASH-WEX alone. Post regimen, the animals were subjected to Rotarod, Narrow Beam Walking and Novel Object Recognition test to analyze their neuromuscular coordination, working memory and learning functions. The rats were then sacrificed to isolate the brain regions and expression of proteins associated with synaptic plasticity and cell survival was studied using Western blotting and Quantitative real time PCR. Further, neuroprotective potential of ASH-WEX and its active fraction (FIV) against inflammatory neurodegeneration was studied and validated using in vitro model system of microglial conditioned medium–treated neuronal cultures and microglial-neuronal co-cultures.

**Results:**

Orally administered ASH-WEX significantly suppressed the cognitive and motor-coordination impairments in rats. On the molecular basis, ASH-WEX supplementation also regulated the expression of various proteins involved in synaptic plasticity and neuronal cell survival. Since microglial-neuronal crosstalk is crucial for maintaining CNS homeostasis, the current study was further extended to ascertain whether LPS–mediated microglial activation caused damage to neurons via direct cell to cell contact or through secretion of inflammatory mediators. ASH-WEX and FIV pretreatment was found to restore neurite outgrowth and protect neurons from apoptotic cell death caused by LPS-induced neuroinflammation in both activated microglial conditioned medium–treated neuronal cultures as well as microglial-neuronal co-cultures.

**Conclusion:**

This extensive study using in vivo and in vitro model systems provides first ever pre-clinical evidence that ASH-WEX can be used as a promising natural therapeutic remedial for the prevention of neurodegeneration and cognitive impairments associated with peripheral inflammation and neuroinflammation.

## Background

Neuroinflammation is considered to be a critical risk factor responsible for impaired neurogenesis, synaptic plasticity and cognitive deficits associated with several neurodegenerative diseases (NDDs) [1]. The prevalence of the neurodegenerative diseases (NDDs) has been increasing significantly with the concurrent increase in the life expectancy globally. Although brain is immunologically privileged, various epidemiological studies have positively correlated the systemic inflammation linked with various chronic inflammatory diseases like T2D, atherosclerosis, rheumatoid arthritis, inflammatory bowel disease, Crohn’s disease, ulcerative colitis etc. with the AD and PD risk [2, 3]. Systemic inflammatory responses signal to the brain through neural route via terminals of vagus nerve and humoral route via circumventricular organs lacking BBB. Inflammatory signals also communicate across the BBB via interactions between the perivascular macrophages and resident microglia through activation of endothelial cells [2, 4]. The microglia, brain immune cells along with glia and neurons are found to be implicated in neuroinflammation linked to pathology of NDDs [5]. They are exquisitely sensitive to endogenous (like stroke, autoimmune processes) and exogenous (e.g. pathogens or psychological stressors) challenges and undergoes phenotypic switch to adopt an activated neurotoxic state. This leads to perturbances in healthy neuron-glial interactions and result in impaired neuronal plasticity and cognitive functions [6].

Activated microglia can induce significant and highly destructive neurotoxic effects directly or indirectly by excessive production of a large array of neurotoxicity mediators which act alone or in combination to damage neurons [7, 8]. Normally microglial cells are neuroprotective i.e. they safeguard the neurons by killing pathogens, removing debris, amyloid β and also provide them with nutritive factors such as BDNF and IGF1. But while attempting to limit infection or when this protective function is not fully accomplished, they accidentally damage the neurons [9, 10]. Co-activation of pro–inflammatory markers and associated cytotoxic products during neuroinflammation are detrimental for neurons by modulating expression of synaptic proteins and their functions. Postmortem brains from bipolar disorder (BD) and Schizophrenic patients showed upregulated neuroinflammatory markers along with decreased synaptic markers (drebin and synaptophysin) and have been linked with cognitive impairments in BD and SZ patients [1]. Microglia mediated neuroinflammation also negatively impacts the neurogenesis by causing impaired neuronal survival and proliferation of the new neurons. Ekdahl et al. (2003) demonstrated that LPS from *E.coli* administered intracortically or intraperitoneally induces strong immune responses in brain and reduces both neurogenesis and neurodifferentiation [11]. The consequences of inflammation on neurogenesis negatively impacts cognition and spatial information [12]. Neuroinflammation also affects the recruitment and correct integration of newborn neurons into hippocampal network encoding spatial information [13]. The current inputs from literature suggest that simultaneously targeting peripheral and central inflammation may be a promising strategy to combat neurodegeneration.

*Withania somnifera* (L.) Dunal (Ashwagandha) has been acknowledged as an adaptogen or rejuvenator in the Ayurvedic medicine from centuries for its potency to normalize physiological functions via regulating the endocrine and immune functions [14, 15]. “Withanolides” inclusive of steroidal alkaloids and lactones are the major pharmacologically active constituents of Ashwagandha extracts prepared from leaves, shoots and roots of plant. Out of these, withaferin A, withanone, withanolide A and withanolide D constitute the major fractions. Withaferin A has been suggested as a natural anti-inflammatory and neuroprotective candidate in various neurological disorders model like spinal cord injury, high-fat diet-induced obesity and several NDDs models [16–18]. The neurite outgrowth promoting activity of the Ashwagandha root methanol extract has been attributed to presence of Withanolide A, withanoside IV, and withanoside VI as the active components [19, 20]. All these active ingredients collectively contribute to neuroprotective activity of Ashwagandha. Majority of the studies on Ashwagandha reported previously in literature have evaluated its neuroprotective activities using alcoholic formulations of roots [21–26]. In this study, we used the leaf based water extract of Ashwagandha to scientifically ascertain the doctrinal use of this Ayurvedic elite, a pharmacological important plant. Moreover, aqueous formulations are easy to prepare and safe, as it avoids the use of organic solvents and scarification of plant is not required.

Ashwagandha leaf water extract (ASH-WEX) and its active fraction (FIV) were previously reported by us to prevent the LPS-induced activation of microglial cells and inhibited the secretion of various pro-inflammatory mediators in primary microglial cultures and BV-2 cell line used as in vitro model system [27]. The anti-neuroinflammatory activity of ASH-WEX and FIV has been ascribed to presence of withaferin A and withanone as revealed by HPLC analysis. Moreover, ASH-WEX was found to prevent systemic lipopolysaccharide (LPS) induced reactive gliosis, expression of pro-inflammatory cytokines and nitro-oxidative stress products. Additionally, NFκB, P38 and JNK MAPKs pathways were found to be the principal molecular mechanism underlies the anti-inflammatory activity of ASH-WEX [28]. Therefore, the objective of current study was to further explore the neuroprotective potential of ASH-WEX to ameliorate cognitive impairments and synaptic plasticity changes induced by systemic LPS associated inflammation using both in vitro and in vivo model system. LPS-induced neuroinflammation has been reported to induce CNS-mediated behavioral responses collectively known as “sickness behavior” which are defined by impaired motor functions, diminished environmental or social exploration along with learning and memory dysfunctions [29]. Therefore, systemic LPS-induced inflammation has been often used in literature to understand molecular mechanisms of cognitive dysfunctions due to inflammation and also to develop targeted remedies for neurological symptoms [3, 30].

The beneficial effect of the ASH-WEX against systemic LPS-induced cognitive deficits was assessed by behavioral tests such as Narrow beam walking test and Novel object recognition (NOR) test and motor coordination by Rotarod test. Further to elucidate the molecular basis of behavioral effects, the expression of various proteins regulating synaptic plasticity and cell survival were studied. Further to investigate the role of microglia in inflammation driven neurotoxicity and neuroprotective activity of ASH-WEX, we used in vitro model system of conditioned media and co-culture regimen. The current study manifestated that ASH-WEX may be potential candidate to ameliorate neurodegeneration and behavioral deficits of learning and memory as well as motor coordination caused by peripheral inflammation.

## Methods

### Animals and experimental conditions

3–4 months old disease free wistar strain male albino rats with body weight ranges from 130 to 150 g were purchased from NIPER, Mohali, Punjab, India and used in the present study. 3–4 animals were housed per cage with free access to food and water under controlled temperature (25 ± 2 °C) and humidity conditions with 12 h light/dark cycle. Protocols were duly approved by Institutional Animal Ethical Committee (IAEC) of the Guru Nanak Dev University, Amritsar, Punjab, India as per Committee for the Purpose of Control and Supervision of Experiments on Animals (CPSCEA) guidelines for care and use of laboratory animals laid down by same committee. All experimental procedures were conducted according to ARRIVE guidelines.

The animals were randomized in four groups:

1. Control: Orally administered with water as vehicle for 8 weeks
2. LPS alone group: Orally administered with water (4 ml/kg body weight) as vehicle for 8 weeks and were injected intraperitoneally with single dose of LPS (5 mg/kg) on last day of treatment regimen.
3. LPS + ASH-WEX group: animals of this group were orally administered with ASH-WEX (4 ml/kg body weight) for 8 weeks and injected with single i.p. dose of LPS (5 mg/kg) on last day.
4. ASH-WEX alone: orally administered with only ASH-WEX (4 ml/kg body weight) for 8 weeks.

12 h after LPS treatment, animals were assessed for their behavior using various behavioral tests like Rotarod, Narrow beam walking test, Novel object recognition test. For detailed biochemical and molecular studies, animals were anesthetized by injecting thiopentone intraperitoneally at the dosage of 1 unit/10 g and decapitated after cervical dislocation to isolate brains. Further, the brains were microdissected to isolate the hippocampus and pyriform cortex (PC) regions of brain.

#### ASH-WEX dosage

Animals were given ASH-WEX orally at the dose of the 4 ml/kg/day extract (equivalent to 140 mg/kg/day dry weight of ASH-WEX) standardized and reported previously by our lab [28, 31–33]. For in vitro studies, ASH-WEX and one of its active fraction (FIV) were used at the concentration of 0.2% and 10 μg/mL, respectively as reported in our previous study [27].

### Behavioral studies

#### Rotarod test

Rotarod test was performed to monitor the motor coordination of the animals (*n* = 8/group). Its apparatus is an automated motor operated treadmill (Rotamex-5; Columbus Instruments). It consists of rotating spindle having dimensions 7.0*9.5 cm for rats with 44.5 cm fall height from the rod center. This test was carried out at a constant speed of 10 rpm on individual animal for 300 s and was recorded for the time spent by the animals on the rotating rod and number of falls from it.

#### Narrow beam walk test

Narrow beam walk test was carried out to evaluate the motor balance and grip strength of the rats (*n* = 8/group) as described by Goldstein and Davis (1990) [34]. The apparatus is equipped with 1 m long and 1–2 cm wide beam placed horizontally at 30 cm elevation above the floor. Rats were allowed to walk across the beam from one end to dark home cage at the other end. The test consists of 2 days training and 1 day testing. The frequency of paw slippage and time taken to cross the beam were recorded. The score was represented as the cumulative of two successful trials on test day and averaged for each animal in a group.

#### Novel object recognition (NOR) test

The NOR test was performed to measure object recognition working memory of animals (*n* = 8/ group) according to protocol as described earlier [35]. Briefly, the test consists of 5 mins empty box habituation of animals for 2 days followed by their 5 mins familiarization with two objects for 3 consecutive days as training program. On the test day, the least preferred object was replaced by novel one and animals were allowed to explore and then assessed for their preference for the objects, exploration time and number of episodes. Further the animals were analyzed for grooming and the rearing behavior in the open field box of NOR test to correlate the effect of stress and anxiety caused by LPS-induced neuroinflammation on memory and cognition. Any grooming bout of duration more than 5 secs was considered as actual grooming bout. The number of grooming episodes and total time spent in grooming were recorded and averaged for each group.

### Western blotting

Western blotting was performed according to protocol as described earlier (Gupta and Kaur 2018) [28]. Briefly the animals (*n* = 4–5/group) were anesthetized by injecting thiopentone intraperitoneally at the dosage of 1 unit/10 g and decapitated after cervical dislocation to isolate brains. Further, the hippocampus and pyriform cortex (PC) regions of brain were microdissected, homogenized and their protein lysates were prepared. Protein concentration was estimated using Bradford method followed by preparation of samples which were then electrophoretically resolved and transferred onto the membranes. After blocking the membranes, they were probed with monoclonal anti-PSA-NCAM (1:1500), anti-NCAM (1:2000), anti-CaMKIIα (1:2000), anti-CaN (1:2000) anti-phospho Akt (1:2000), anti-MAP2 (1:2000), anti-GAP43 (1:2500), (Sigma-Aldrich St. Louis, MO, USA), anti-TrkB (1:1000) (Trk A & B antibody sampler kit, Cell Signaling Technology, MA, USA), anti-Bcl-xL (1: 1500) for overnight at 4 °C. Further, the membranes were washed and incubated with their respective secondary antibodies (Genei) HRP-labeled anti-mouse IgM (1:2000) for PSA-NCAM, anti-rabbit IgG (1:5000) for pAkt1, anti-rabbit IgG (1:1500) for anti-TrkB, anti-mouse IgG (1:5000) for CaMKIIα, CaN, MAP2, GAP43 (Merck Millipore, USA) for 2 h at 25 °C. α-Tubulin was used as internal control for protein loading. ECL Prime Western Blotting Detection Reagent was used for visualizing immunoreactive bands using Image Quant LAS 4000 system (GE Healthcare, Little Chalfont, UK). For western blots analysis, the expression of each control (in triplicates) was taken as 100% and then percentage change in expression of protein of interest in test samples compared to corresponding control was calculated. The average and corresponding standard error was then calculated using MS Excel and Sigma Stat. Standard error bars were also plotted on the scale of 100. This way of western blot analysis is well accepted and reliable one. Researchers usually present the relative expression in their test samples either in ratio of control (at the scale of 1) [36–38] or percent of control (at the scale of 100) [39–43]. We have been presenting our western data in previous lab publications in this pattern [27, 28, 32, 33, 44]. Data was analyzed for change in protein expression.

### Quantitative real time PCR (RT-PCR) for mRNA expression analysis

mRNA expression analysis was done according to protocol as described earlier [28]. Briefly, the total RNA was extracted from brain tissues using TRI reagent (Sigma-Aldrich, St. Louis, MO, USA) according to manufacturer’s instructions (*n* = 4–5/group). Further, the RNA was reverse transcribed using M-MLV reverse transcriptase to prepare the cDNA. Amplification of gene of interest was then done from 50 μg of cDNA using StepOne Plus Real Time PCR system (Applied Biosystems, Foster City, CA, USA) according to SYBR chemistry. GAPDH was used as an internal control. The ‘Livak method’ was applied to calculate the relative gene expression and data was presented as 2 ^− ΔΔCt^ ± SEM.

### Primary microglial cultures

Microglial enriched cultures were obtained from primary mixed glial cultures from 1-day-old pups using mild trypsinization method [45] with slight modification as described earlier [27]. Isolated microglial cells were seeded in multiwell plates by trypsinization with 0.25% trypsin-EDTA for 10 mins and treated after 24 h of seeding. For preparation of the microglial conditioned medium, primary microglial cells were pretreated with 0.2% and 10 μg/mL of ASH-WEX and FIV, respectively for 2 h prior to activation with 100 ng/mL LPS [LPS (rough strains) from *E. coli* F583 (Rd mutant)]. The microglial conditioned medium was harvested after 36 h of treatment, centrifuged to make cell free and used freshly for different experiments. Conditioned medium from different groups were designated as CM-Control, CM-ASH-WEX, CM-FIV, CM-LPS, CM-LPS + ASH-WEX, CM-LPS + FIV.

### Primary neuronal cultures

Primary neuronal cultures were prepared from 1-day-old (cortical), 2-day-old (hippocampal) and 7-day-old (cerebellar) pups, respectively using the procedure followed by Frandsen and Schousboe (1990) with some modifications [46]. Briefly, the pup brain was dissected to collect cortex, hippocampus and cerebellum regions; their meninges were carefully removed and the tissue was digested with 0.025% (w/v) trypsin-EDTA solution containing 0.1% DNase for 15 mins at 37 °C. Activity of trypsin was stopped by adding equal amount of trypsin inhibitor solution or media and centrifuged (1000 rpm, 4 mins). The tissue was titurated and the cells were resuspended in the Neurobasal medium. Then the cells were seeded on poly-L-lysine coated 24 well culture plates and maintained in Neurobasal medium (supplemented with bFGF and B12 supplement) at 37 °C in 5% humidified atmosphere.

### Primary microglial-neuronal co-culture

For primary microglial–cerebellar neurons co-culture, firstly the primary microglia cultures were prepared from 1-day-old pups as described above. Microglial cells obtained were seeded at a density of 15,000 cells/mL on PLL coated coverslips. After 24 h, microglial cells were pretreated with ASH-WEX and FIV for another 24 h. Then ASH-WEX and FIV containing medium was removed and cerebellar neurons were cultured from 7-day-old pups as detailed above and seeded on the top of the microglial monolayer in a ratio of 2:1 (neuronal: microglial ratio). After 4 h of seeding cerebellar neurons, microglial-neuronal cultures were challenged with 100 ng/ml LPS for 36 h. Microglial-neuronal co-cultures were then used for MAP2 immunostaining and DAPI nuclear staining to determine the percentage apoptotic cell death. Images captured for MAP2 immunostaining were used to determine the percentage neurite outgrowth. Experiments were performed in triplicate. Neurite lengths were determined from at least 100 cells per group using Image Pro Plus software (Media Cybernetics, Silver Spring, USA) and then analysis was done using Microsoft excel and Sigma Stat for windows (version 3.5).

### Immunocytochemistry

The control and treated cells were washed with 1X PBS thrice, fixed with 1:1 acetone and methanol followed by permeabilization with 0.3% Triton X-100 in PBS (PBST). After blocking with 2% BSA in PBS, cells were incubated with anti-MAP2 (1:200, Sigma-Aldrich) monoclonal antibody diluted in blocking solution for 24 h at 4 °C in a moist chamber. Then the cells were washed twice or thrice with 0.1% PBST followed by incubation with anti-mouse IgG 488 secondary antibody (diluted at a concentration of 1:500 in 2% BSA) for 2 h at room temperature. For nuclear staining, cells were incubated with 1:5000 dilution of DAPI in 1X PBS for 10 mins. Cells were then mounted with anti-fading reagent (Fluoromount, Sigma-Aldrich). Nikon AIR Confocal Laser Microscope was used for capturing the images. Experiments were performed in triplicate. For quantifying MAP2 relative optical intensity, ROIs were selected from different cells (at least 100 cells per group) and analyzed for the intensity using NIS elements AR analysis software version 4.11.00. For neurite outgrowth analysis, at least 100 cells per group were measured for length of neurites using the Image Pro Plus software (Media Cybernetics, Silver Spring, USA) and percentage neurite outgrowth per group was calculated using Microsoft excel and Sigma Stat for windows (version 3.5).

### Statistical analysis

The mean ± SEM of values calculated from minimum of three independent experiments were used to express data. Student’s t-test, one-way ANOVA (Holm-Sidak post hoc method) and two-way ANOVA have been employed to assess the level of significance by using Sigma Stat software for Windows (version 3.5). Values showing *p* ≤ 0.05 were acceptable as statistically significant**.** Only where the significance level comes ≤ 0.05 while comparing different groups, the symbol representing significance has been entered in the graphs.

## Results

### ASH-WEX pretreatment improved neuromuscular coordination

**Fig. 1:**
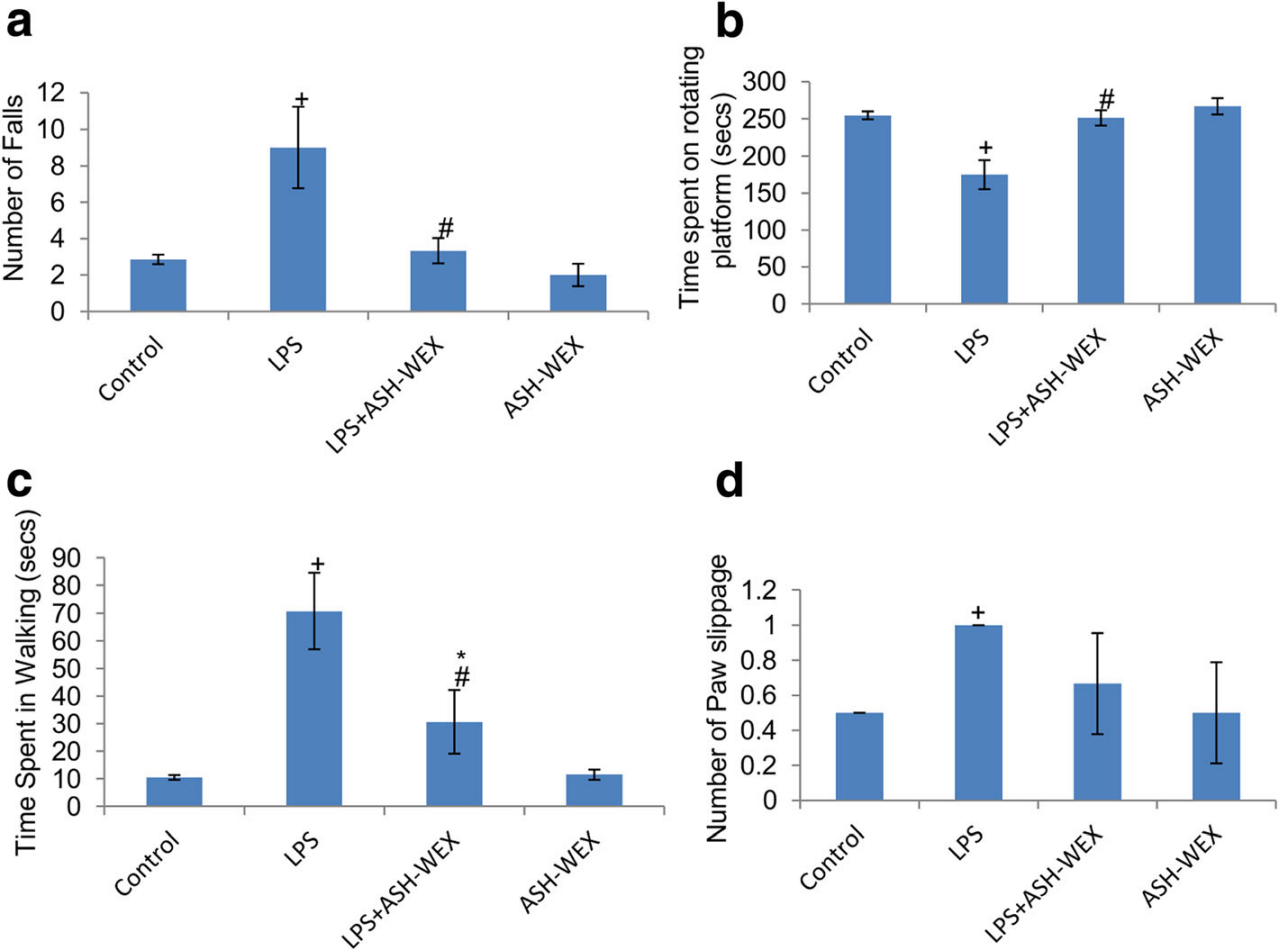
ASH-WEX improves the locomotor coordination impaired due to LPS-induced sickness behavior During rotarod test (*n* = 8/group), ASH-WEX supplemented LPS (LPS + ASH-WEX)-treated rats exhibited better performance than LPS-treated animals as evaluated by their (**a**) latency to fall and (**b**) time spent on rotating rod. ASH-WEX administration also improved the performance of LPS + ASH-WEX rats in narrow beam walking test which was adversely affected by LPS as assessed by (**c**) time taken and (**d**) paw slippage to cross the beam thus implying their intact motor functions. Values are expressed as mean ± SEM. *p ≤ 0.05 control versus ASH-WEX alone and LPS + ASH-WEX groups. ^+^*p* ≤ 0.05 control versus LPS alone group. ^#^*p* ≤ 0.05 LPS alone versus LPS + ASH-WEX group (one-way ANOVA with Holm-Sidak post hoc test)

Animals were examined for their motor coordination using rotarod. LPS injected rats exhibited maximum number of falls (Fig. 1a; *p* ≤ 0.01) and spent significantly less time (Fig. 1b; *p* ≤ 0.001) on rotating rod in comparison to other three groups. However, LPS + ASH-WEX- and ASH-WEX alone-treated rats exhibited significantly lower number of falls (Fig. 1a; *p* ≤ 0.01) and spent more time (Fig. 1b; *p* ≤ 0.001) on rotating rod as compared to LPS alone-treated group. To further confirm the improvement in locomotor activity by ASH-WEX, animals were assessed for the time taken to transverse the narrow beam and the number of paw slippage using narrow beam walk test. LPS-treated rats exhibited maximum number of paw slippages (Fig. 1d; *p* ≤ 0.05) and also took more time (Fig. 1c; *p* ≤ 0.01) to transverse the beam compared to control group. LPS + ASH-WEX- and ASH-WEX alone-treated rats swiftly crossed the beam (Fig. 1c; *p* ≤ 0.01) with least number of paw slippages (Fig. 1d).

### ASH-WEX suppressed LPS-induced cognitive impairments

**Fig. 2:**
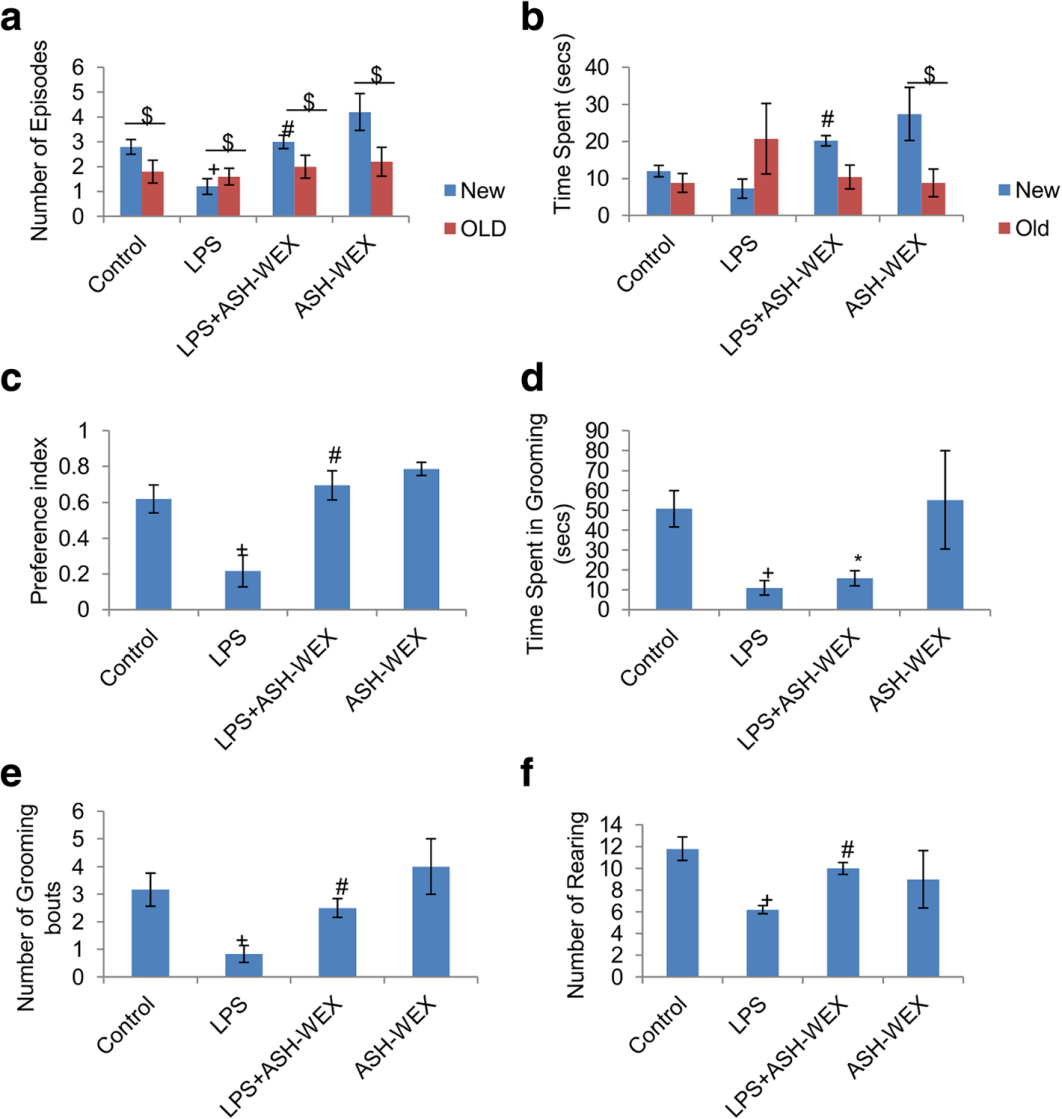
ASH-WEX improves working memory and learning functions Performance of animals (n = 8/group) in Novel object recognition test was presented in panel a-f. ASH-WEX supplemented LPS (LPS + ASH-WEX)-treated rats exhibited more (**a**) number of episodes and (**b**) time spent in exploration of new object than the old one as compared to LPS-treated rats. (**c**) Histogram presenting significantly higher preference index of LPS + ASH-WEX-treated animals for new object over the old one indicates their intact recognition memory. (**d**, **e**, **f**) Histograms depicting the number and duration of the grooming bouts and number of rearings among the different treated groups. Values are expressed as mean ± SEM. *p ≤ 0.05 control versus ASH-WEX alone and LPS + ASH-WEX groups. ^+^*p* ≤ 0.05 control versus LPS alone group. ^#^*p* ≤ 0.05 LPS alone versus LPS + ASH-WEX group. $ represents statistically significant difference within groups (New vs. Old in each group) (one-way ANOVA with Holm-Sidak post hoc test, two-way ANOVA in **a**, **b**)

Further, the animals were tested for their recognition learning and memory using NOR test (Fig. 2). LPS injected rats were observed to show minimum frequency of episodes and total time spent in exploring novel object over the old one implying their impaired memory in recognizing novel object in comparison to other three groups (Fig. 2a and b). In contrast, LPS + ASH-WEX- and ASH-WEX alone-treated rats exhibited normal and intact memory as evident from more frequency of episodes and time spent in exploring novel object over the old one (Fig. 2a and b). Moreover, preference index (PI) score of these animals was more than 0.5 indicating their higher preference for novel object over the old one (Fig. 2c; *p* ≤ 0.001), further approving their normal working and recognition memory. While, PI score for the LPS-treated animals was less than 0.5 suggesting their preference for old over the new object (Fig. 2c; *p* ≤ 0.001).

Moreover, LPS alone-treated rats exhibited longer latency to start grooming with reduced number of grooming bouts and spent less time in grooming during NOR test. Impaired grooming behavior probably could be due to sickness induced by LPS (Fig. 2d and e; *p* ≤ 0.005). LPS + ASH-WEX- and ASH-WEX alone-treated rats exhibited normal grooming behavior with more duration and frequency of grooming bouts comparable to control rats. Similarly, LPS-treated animals exhibited less number of rearing than control group animals, while, LPS + ASH-WEX- and ASH-WEX alone-treated animals exhibited maximum number of rearing implying their interest in exploration of empty box and reduction in LPS-induced stress by ASH-WEX administration (Fig. 2f; *p* ≤ 0.001).

### ASH-WEX and the expression of synaptic plasticity and neuronal survival proteins

**Fig. 3:**
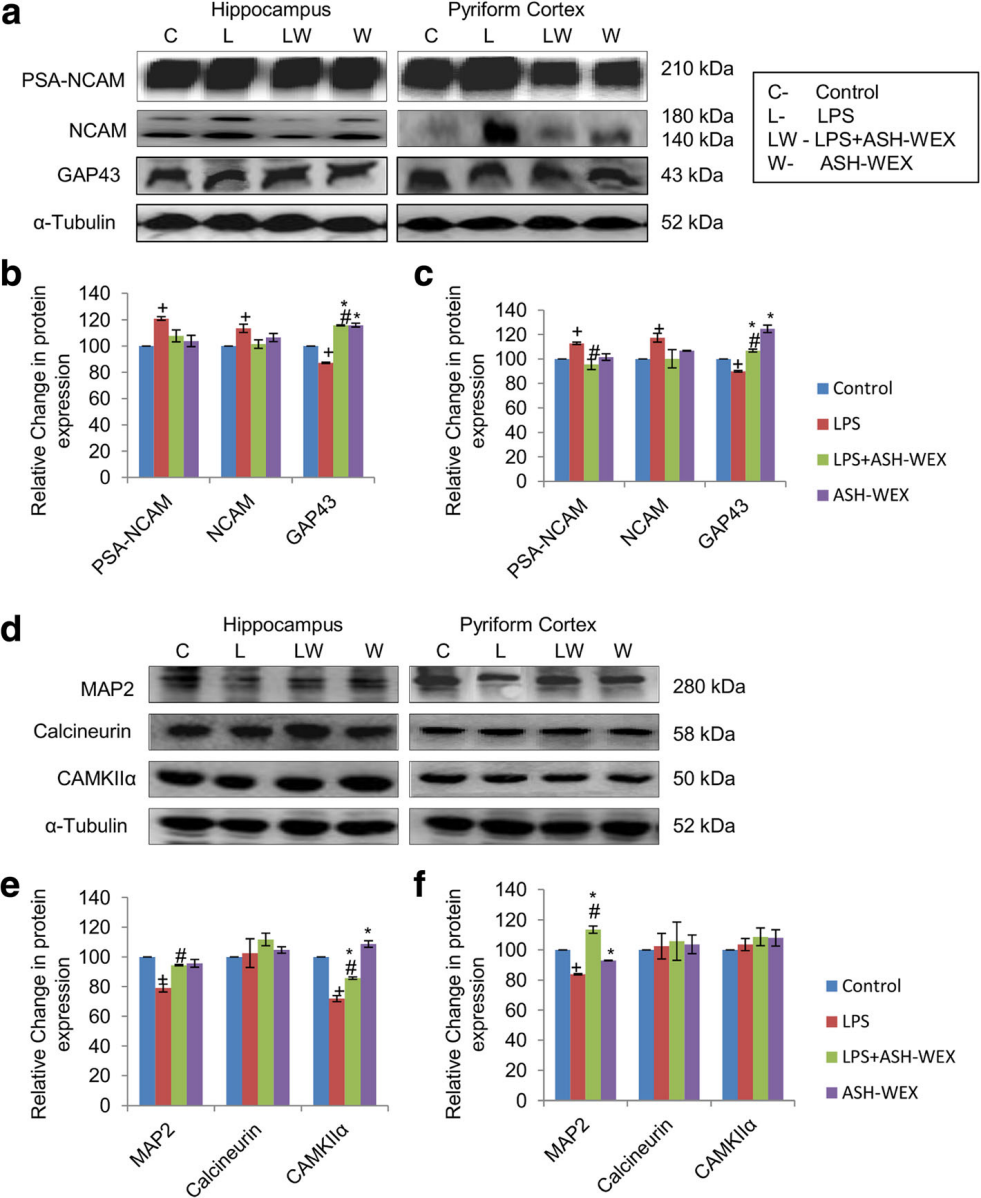
ASH-WEX normalizes the expression of various synaptic plasticity markers (**a**) Representative western blots of the PSA-NCAM, NCAM and GAP43 in hippocampus (left panel) and pyriform cortex (right panel) regions of brain amidst the four group of animals (*n* = 4–5/group). (**b**, **c**) histograms depicting relative densitometric analysis amidst the different treatment groups from (**b**) hippocampus and (**c**) pyriform cortex regions of brain plotted as Mean ± SEM (taking control group as 100%). (Expression of NCAM was calculated as cumulative of both 180 kDa and 140 kDa bands) (**d**) Representative immunoblots of MAP2, Calcineurin and CAMKIIα in hippocampus (left panel) and pyriform cortex (right panel) regions of brain amidst the four group of animals (n = 4–5/group). (**e**, **f**) Histograms depicting relative densitometric analysis amidst the different treatment groups in (**e**) hippocampus and (**f**) pyriform cortex regions plotted as Mean ± SEM (taking control group as 100%). α-Tubulin was used for the normalization of protein expression. *p ≤ 0.05 control versus ASH-WEX alone and LPS + ASH-WEX groups. ^+^*p* ≤ 0.05 control versus LPS alone group. ^#^*p* ≤ 0.05 LPS alone versus LPS + ASH-WEX group (one-way ANOVA with Holm-Sidak post hoc test)

LPS treatment upregulated the expression of PSA-NCAM as compared to control rats, whereas its expression in LPS + ASH-WEX- and ASH-WEX alone-treated rats were near to control level in both hippocampus (Fig. 3a and b; *p* ≤ 0.05) and PC (Fig. 3a and c; *p* ≤ 0.05) regions of the brain. Similar changes were also observed in the expression of NCAM in both regions (Fig. 3a-c; *p* ≤ 0.05). LPS treatment also decreased the expression of neuronal growth related protein GAP43 expression up to 87.2 and 90% in hippocampus and PC regions, respectively as compared to control rats (taken as 100%). Whereas, its expression was significantly upregulated up to 116% (hippocampus) and 107% (PC) in LPS + ASH-WEX-treated rats (Fig. 3a-c; *p* ≤ 0.05). GAP43 expression was also near control level in both brain regions of ASH-WEX alone-treated rats. Furthermore, the expression of the neuronal structural protein MAP2 was normalized by ASH-WEX supplementation which was downregulated by LPS treatment in both brain regions (Fig. 3d-f; *p* ≤ 0.05).

Further, the expression of Ca^2+^ and calmodulin dependent serine threonine protein phosphatase (calcineurin) and protein kinase (CAMKIIα) were studied in hippocampus and PC regions of brain which play an integral role in synaptic transmission modulation. LPS significantly reduced CAMKIIα expression (up to 71%) as compared to control rats (taken as 100%) while its expression was restored up to 86% in LPS + ASH-WEX-treated rats compared to LPS alone group in hippocampus region (Fig. 3d and e; *p* ≤ 0.05). In PC region, no significant variations were found in CAMKIIα expression amidst all the four group of animals (Fig. 3d and f; *p* ≤ 0.05). Its expression remained normal in both brain regions of ASH-WEX alone-treated animals. In contrast, the expression of calcineurin was not altered among all groups in hippocampus (Fig. 3d and e) and PC (Fig. 3d and f) regions of brain.

### ASH-WEX and BDNF-TrkB receptor/ PI3K-Akt pathway

**Fig. 4:**
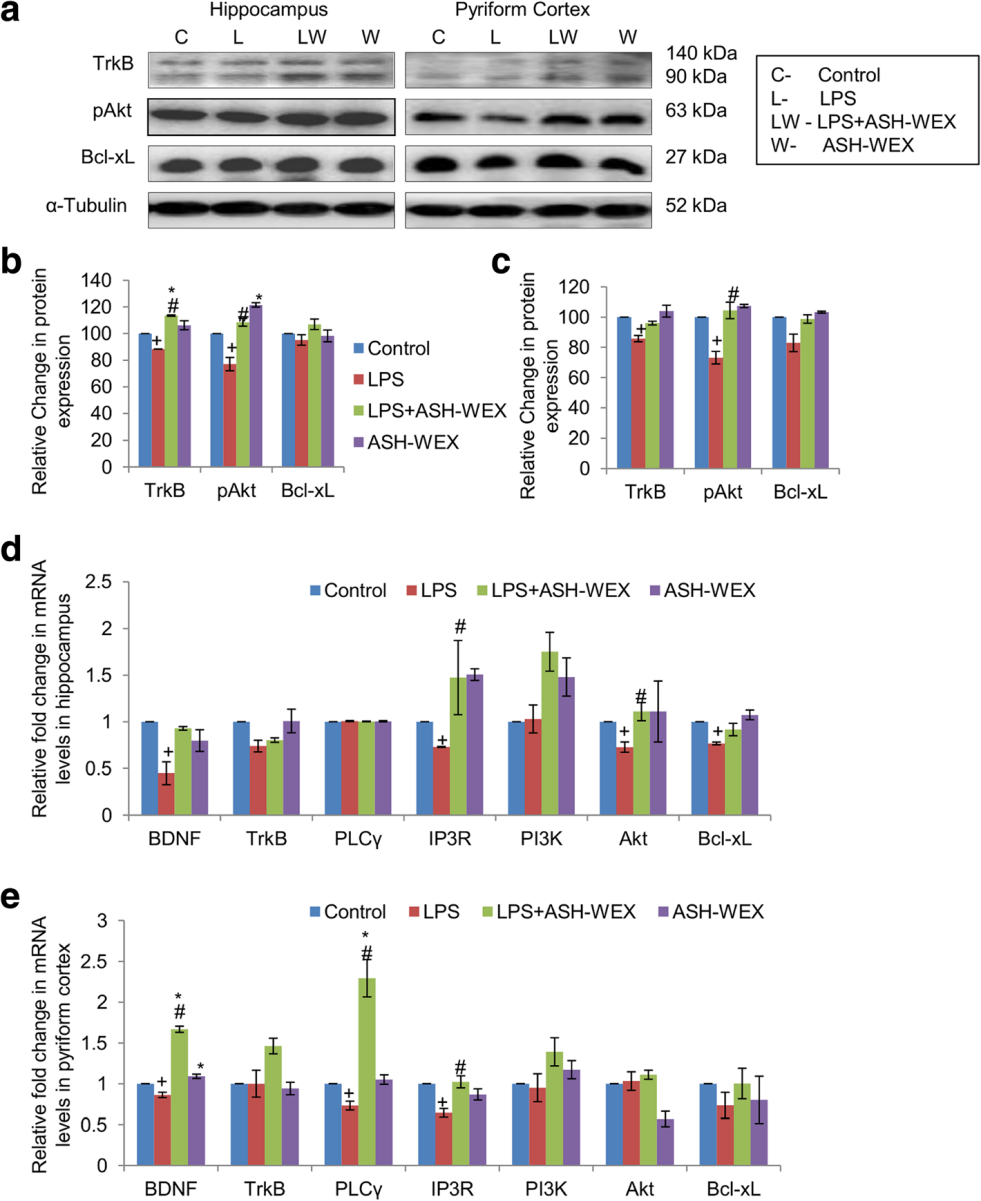
ASH-WEX restores BDNF–TrkB signaling proteins (**a**) Representative immunoblots of the TrkB, pAkt, Bcl-xL in hippocampus (left panel) and pyriform cortex (right panel) regions of brain amidst the four group of animals (n = 4–5/group). (**b**, **c**) histograms presenting relative densitometric analysis in (**b**) hippocampus and (**c**) pyriform cortex regions amidst the different treatment groups plotted as Mean ± SEM (taking control group as 100%). α-Tubulin was used for the normalization of protein expression. (**d**, **e**) Histograms depicting relative mRNA levels of BDNF, TrkB, PLCγ, IP3R, PI3K, Akt, and Bcl-xL in (**d**) hippocampus and (**e**) pyriform cortex regions amidst the four groups (n = 4–5/group) plotted as Mean ± SEM from three independent experiments. *p ≤ 0.05 control versus ASH-WEX alone and LPS + ASH-WEX groups. ^+^*p* ≤ 0.05 control versus LPS alone group. ^#^*p* ≤ 0.05 LPS alone versus LPS + ASH-WEX group (one-way ANOVA with Holm-Sidak post hoc test)

The neurotrophin BDNF plays important role in neurons proliferation and survival by binding to TrkB receptor to initiate signaling pathway. At transcriptional level, LPS + ASH-WEX- and ASH-WEX alone-treated rats showed near control level expression of BDNF and TrkB in both hippocampus and PC regions which was significantly downregulated after LPS treatment (Fig. 4d and e; *p* ≤ 0.05). mRNA expression of BDNF was significantly upregulated up to 1.7 fold in PC region of LPS + ASH-WEX-treated animals (Fig. 4e; *p* ≤ 0.05). Expression of TrkB was also seen to be normalized significantly by ASH-WEX supplementation at translational level in both hippocampus (Fig. 4a and b; *p* ≤ 0.05) and PC (Fig. 4a and c; *p* ≤ 0.05) regions. The phosphotyrosine residues of TrkB receptor act as binding sites for specific cytoplasmic signaling and scaffolding proteins that in turn activate PI3K-Akt pathway. The expression of PI3K although did not change in LPS alone-treated animals compared to control group but its expression was found to be markedly upregulated in LPS + ASH-WEX and ASH-WEX alone rats from both hippocampus (Fig. 4d) and PC (Fig. 4e) regions. ASH-WEX supplementation also normalized the mRNA expression of total Akt which was significantly reduced by LPS treatment in hippocampus region (Fig. 4d; *p* ≤ 0.05). However, Akt mRNA expression did not altered among all groups in PC region (Fig. 4e). Similarly, LPS-induced decrease in expression of phosphorylated Akt was also recovered in LPS + ASH-WEX- and ASH-WEX alone-treated rats from both brain regions (Fig. 4a-c; *p* ≤ 0.05).

Akt further binds to BAD protein (BCL2 associated death promoter) and causes its inactivation. BAD protein complexes with 14-3-3 proteins in cytoplasm which prevents association with mitochondrially localized Bcl-xL and therefore inhibits apoptosis. LPS treatment marginally reduced Bcl-xL expression up to 95 and 82% from hippocampus and PC regions, respectively in comparison to control rats (taken as 100%), whereas, ASH-WEX administration restored the expression of Bcl-xL up to 107 and 99% in hippocampus and PC regions, respectively (Fig. 4a-c; *p* ≤ 0.05). ASH-WEX alone-treated rats exhibited near control level expression of Bcl-xL in both brain regions. mRNA expression of Bcl-xL was observed to be near control level in LPS + ASH-WEX- and ASH-WEX alone-treated animals but was significantly reduced with LPS treatment from hippocampus region (Fig. 4d; *p* ≤ 0.05). In PC region, no significant variations were found in mRNA levels of Bcl-xL among all the groups (Fig. 4e; *p* ≤ 0.05).

BDNF-TrkB pathway also activates PLCγ signaling pathway which further mediates the signals through IP3 by binding to its receptor and causing rapid release of Ca^2+^ involved to regulate neuronal activity and synaptic transmission. In PC region, ASH-WEX treatment showed 2-fold increase in expression of PLCγ which was decreased significantly in LPS alone-treated animals (Fig. 4e; *p* ≤ 0.05). mRNA expression of PLCγ remained unaffected among all groups in hippocampus region (Fig. 4d). LPS treatment significantly reduced the mRNA levels of IP3R gene in both hippocampus and PC regions, whereas, its expression was significantly upregulated by ASH-WEX administration (Fig. 4d and e; *p* ≤ 0.05).

### ASH-WEX and FIV protected neurons against microglial mediated neurotoxicity

**Fig. 5:**
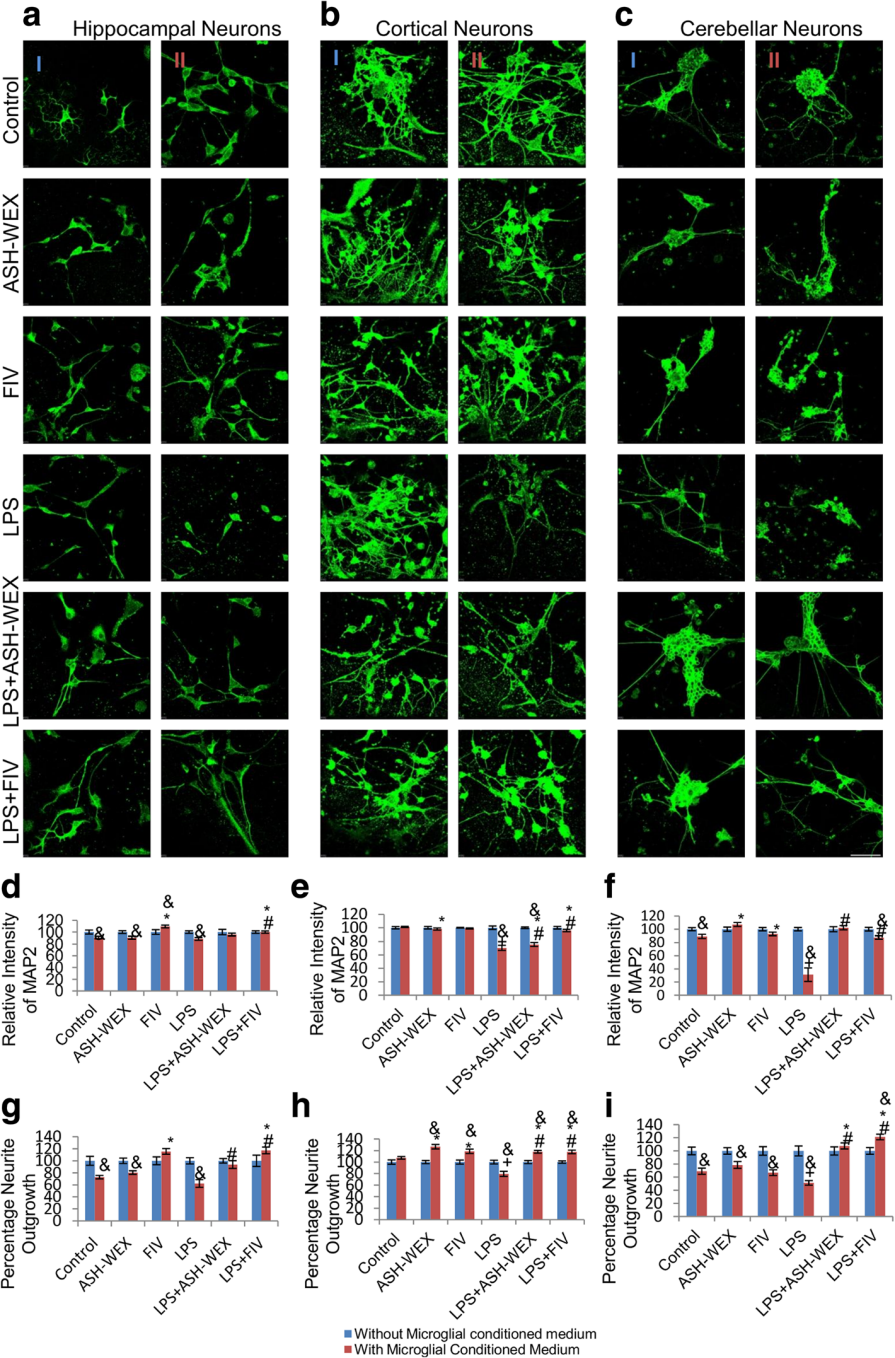
ASH-WEX and FIV protect neurons from LPS activated microglial mediated neurotoxicity (**a**, **b**, **c**) Representative Confocal micrographs of MAP2 immunostaining of primary hippocampal, cortical and cerebellar neurons, respectively treated with (column I) ASH-WEX and FIV with or without activation with 100 ng/mL LPS and (column II) LPS conditioned medium collected from LPS stimulated primary microglial cells with or without pretreatment with ASH-WEX and FIV for 24 h. (**d**, **e**, **f**) Histograms presenting relative optical intensity of MAP2 (Mean ± SEM) in primary hippocampal, cortical and cerebellar neurons, respectively,among different treatment groups. (**g**, **h**, **i**) Histograms depicting percentage neurite outgrowth (Mean ± SEM) in primary hippocampal, cortical and cerebellar neurons, respectively, among different treatment groups calculated from three independent experiments. Cultures treated directly with ASH-WEX and FIV with or without activation with LPS were taken as 100%. Direct treatment of 100 ng/mL LPS was observed to be non-toxic for neurons. *p ≤ 0.05 CM-Control versus CM-ASH-WEX alone, CM-FIV alone, CM-LPS + ASH-WEX and CM-LPS + FIV-treated groups. ^+^*p* ≤ 0.05 CM-Control versus CM-LPS-treated group. ^#^*p* ≤ 0.05 CM-LPS- versus CM-LPS + ASH-WEX- and CM-LPS + FIV-treated groups. ^&^*p* ≤ 0.05 represents statistically significant difference between cultures treated with or without microglial conditioned medium corresponding to different treatment groups (one way ANOVA with Holm-Sidak post hoc test). Images were captured using Nikon A1R Laser Scanning Confocal Microscope at 60X magnification (Scale bar = 50 μm)

To further validate the neuroprotective effect of ASH-WEX and FIV against LPS-induced neuroinflammation, cell free conditioned medium (CM) of microglial cultures was prepared and tested (as per details in material and methods section). Direct treatment of LPS at the concentration of 100 ng/mL was found to be non toxic to neurons, whereas, neuronal cultures treated with CM-LPS showed significant downregulation in expression of MAP2 (Fig. 5a-c column II) as compared to CM-Control (Fig. 5a-c column II) and LPS alone-treated neurons (Fig. 5a-c column I). In contrast, treatment of neuronal cultures with CM-LPS + ASH-WEX and CM-LPS + FIV (Fig. 5a-c column II) showed near normal expression of MAP2 as compared to LPS + ASH-WEX- and LPS + FIV-treated neurons (Fig. 5a-c column I) as depicted by MAP2 immunostaining (Fig. 5a-c) and their intensity plots (Fig. 5d-f; *p* ≤ 0.05). MAP2 expression remained near control level in neurons treated with CM-ASH-WEX, CM-FIV, ASH-WEX and FIV alone. Since, neuroinflammation impairs the axonal regrowth and contributes to lack of neural regeneration, so confocal images of MAP2 immunostaining were further analysed for percentage neurite outgrowth (Fig. 5g-i). CM-LPS treatment group showed significant degeneration of neurite outgrowth as compared to CM-Control- and LPS alone-treated cortical, hippocampal and cerebellar neuronal cultures (Fig. 5g-i; *p* ≤ 0.05). CM-LPS + ASH-WEX and CM-LPS + FIV treatment also restored the neurite outgrowth as shown in histograms (Fig. 5 g-i).

### ASH-WEX and FIV prevented the neuronal apoptosis against microglial mediated inflammatory injury

**Fig. 6:**
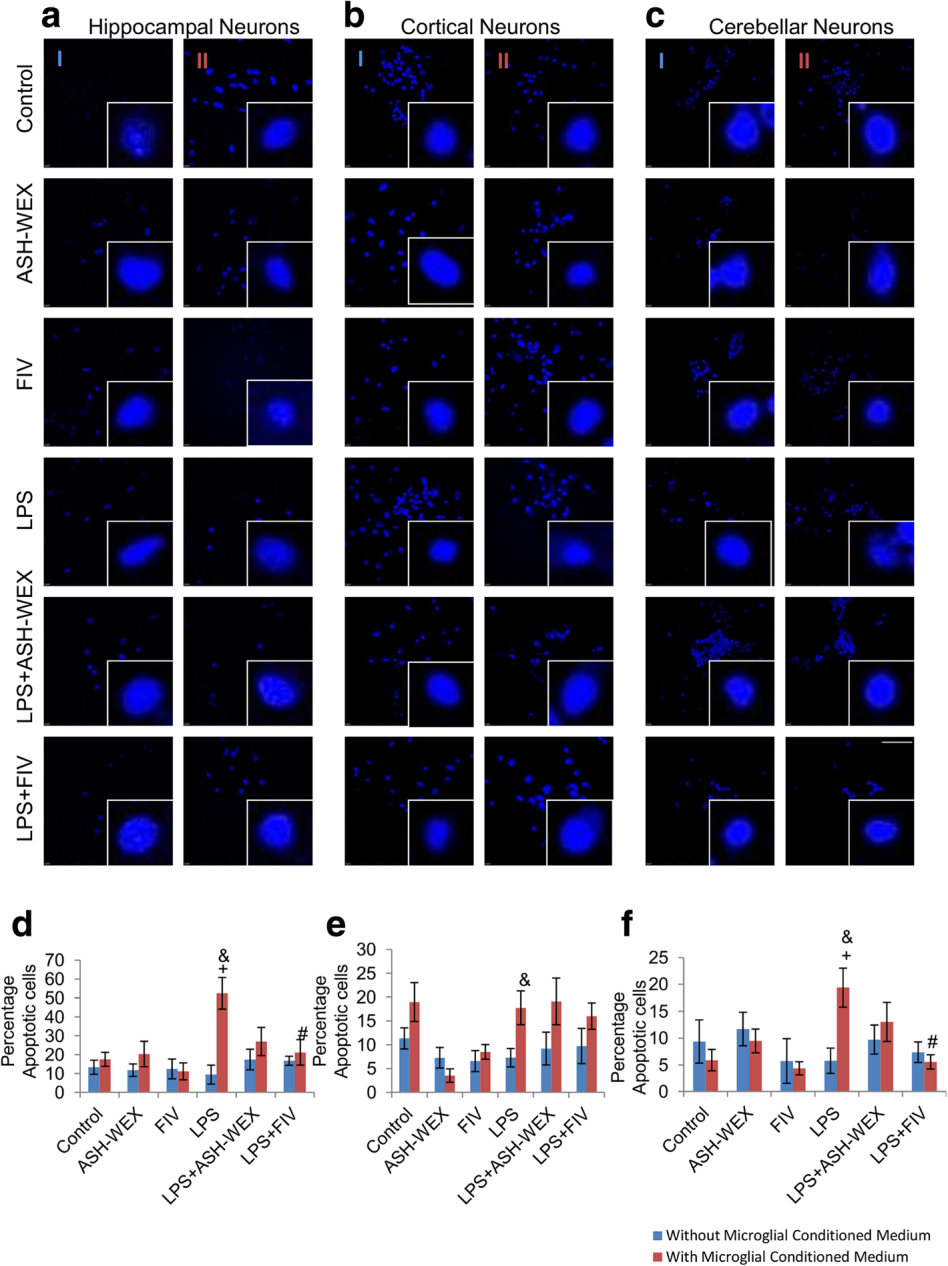
ASH-WEX and FIV prevent apoptosis of neurons induced by LPS-induced neuroinflammation (**a**, **b**, **c**) Representative Confocal micrographs of DAPI immunostaining of primary cortical, hippocampal and cerebellar neurons respectively treated with (column I) ASH-WEX and FIV directly with or without activation with 100 ng/mL LPS, (column II) conditioned medium collected from LPS stimulated primary microglial cells with or without pretreatment with ASH-WEX and FIV for 24 h. (**d**, **e**, **f**) Histograms depicting percentage of apoptotic cells (Mean ± SEM) in primary hippocampal, cortical and cerebellar neuronal cultures, respectively, among different treatment groups calculated from three independent experiments. Cultures treated directly with ASH-WEX and FIV with or without activation with LPS were taken as 100%. *p ≤ 0.05 CM-Control versus CM-ASH-WEX alone, CM-FIV alone, CM-LPS + ASH-WEX and CM-LPS + FIV-treated groups. ^+^*p* ≤ 0.05 CM-Control versus CM-LPS-treated group. ^#^*p* ≤ 0.05 CM-LPS- versus CM-LPS + ASH-WEX- and CM-LPS + FIV-treated groups. ^&^*p* ≤ 0.05 represents statistically significant difference between cultures treated with or without microglial conditioned medium corresponding to different treatment groups (one way ANOVA with Holm-Sidak post hoc test). Images were captured using Nikon A1R Laser Scanning Confocal Microscope at 60X magnification (Scale bar = 50 μm)

Apoptosis was determined by analyzing nuclear morphology using 4′,6′-diamidino-2-phenylindole (DAPI) staining (Fig. 6). 4, 6-Diamidino-2-phenylindole (DAPI) is a DNA binding dye and is commonly used assay for detection of apoptosis in cells. Apoptotic cells have more permeability for dye so these cells show more intense blue fluorescence due to chromosomal condensation, whereas, normal cells show uniformly stained round nucleus with clear margins. So DAPI staining can be used to observe differences between apoptotic and non-apoptotic cells based on the intensity of fluorescence and the conformation of nucleus. Neurons treated with CM-LPS for 24 h showed both disintegration and condensation of nuclei resembling apoptotic cell death (Fig. 6a-c) and caused significant apoptosis of neurons as indicated by highest percentage of apoptotic cells (Fig. 6d-f; *p* ≤ 0.05). CM-LPS + ASH-WEX and CM-LPS + FIV treatment effectively suppressed apoptosis of neurons. CM-ASH-WEX, CM-FIV, ASH-WEX and FIV alone treatment did not affect the survival of the neurons. However, CM-LPS + ASH-WEX and CM-LPS + FIV treatment restored the neuronal cell viability thus suggesting the neuroprotective effect of ASH-WEX and FIV (Fig. 6).

### ASH-WEX and FIV also protected the neurons in microglial-neuronal co-culture

**Fig. 7:**
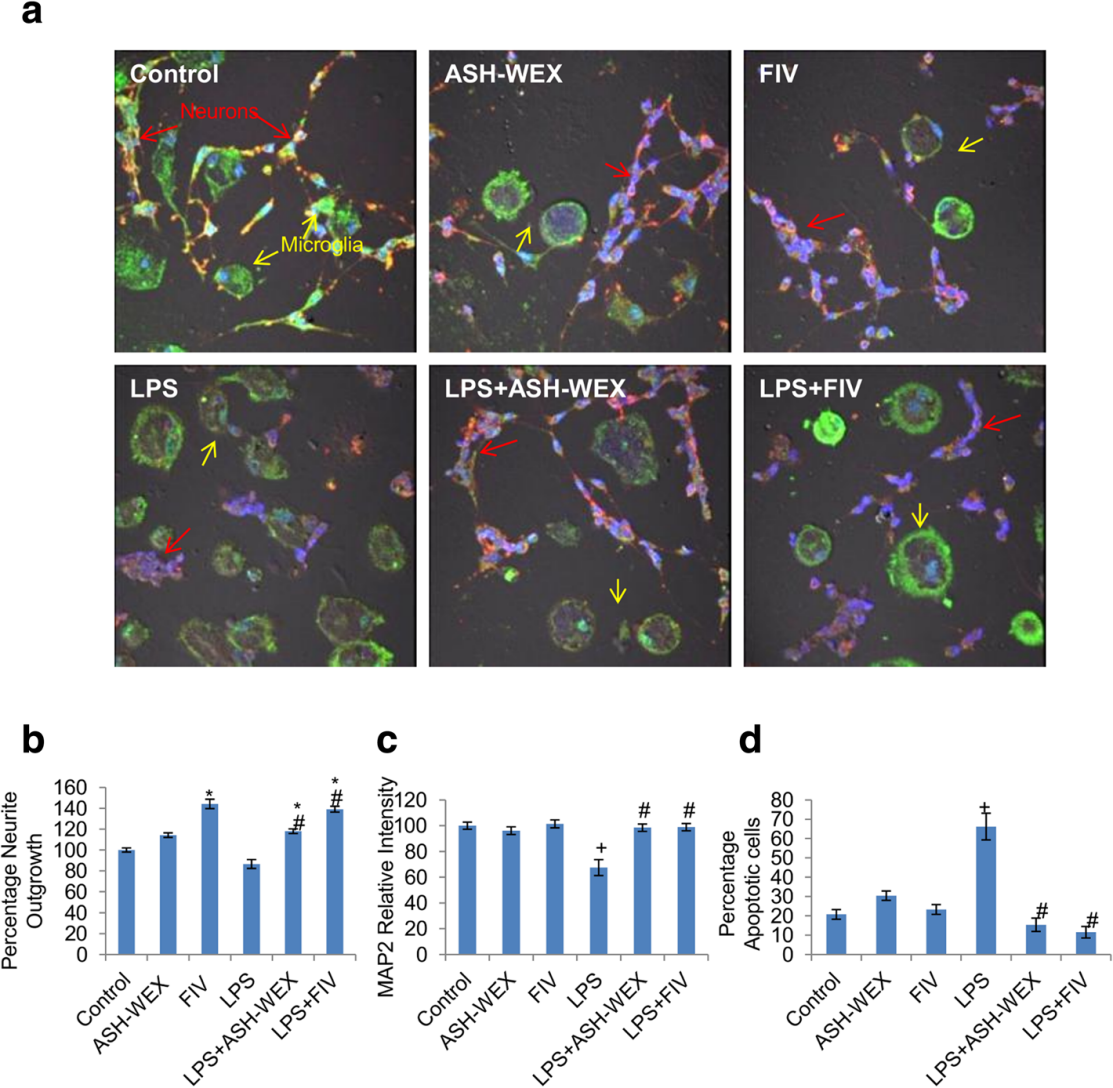
ASH-WEX and FIV also inhibit inflammatory neurodegeneration in microglial-neuronal co-culture (**A**) Representative Confocal micrographs of MAP2 (red) and Iba-1 (green) co-immunostaining in cerebellar neurons co-cultured with microglia pretreated with 0.2% ASH-WEX and 10 μg/mL FIV for 24 h, then challenged with 100 ng/mL LPS for 36 h. Nuclei were stained with DAPI. Red arrows denote cerebellar neurons and yellow arrows denote microglial cells. (**b**, **c**, **d**) Histograms presenting the data of percentage neurite length, relative optical intensity of MAP2 and percentage of apoptotic cells (Mean ± SEM) among the different treatment groups calculated from three independent experiments. *p ≤ 0.05 untreated control versus ASH-WEX alone, FIV alone, LPS + ASH-WEX and LPS + FIV-treated groups. ^+^*p* ≤ 0.05 control versus LPS-treated group. ^#^*p* ≤ 0.05 LPS- versus LPS + ASH-WEX- and LPS + FIV-treated groups (one-way ANOVA with Holm-Sidak post hoc test). Images were captured using Nikon A1R Laser Scanning Confocal Microscope at 60X magnification (Scale bar = 50 μm)

To further investigate whether microglia directly participate in neurotoxicity via cell to cell contact or only through secreted chemical mediators of inflammation, we performed the primary microglia-cerebellar neurons co-culture and analyzed for MAP2 neuronal protein expression, percentage neurite outgrowth and percentage neuronal apoptotic cell death (Fig. 7). Microglial-neuronal co-cultures treated with LPS alone exhibited less number of MAP2 positive neurons as well as (up to 67%) reduced MAP2 expression (Fig. 7c; *p* ≤ 0.05) and showed neuronal degeneration with retracted neurite outgrowth (Fig. 7b) as compared to control co-cultures. Further, the proportion of the cerebellar neurons showing apoptosis like nuclear condensation reached up to 66% (Fig. 7d; *p* ≤ 0.05) of the total neuronal population after 36 h of co-culture. In contrast, ASH-WEX and FIV treatment normalized MAP2 expression, sustained neuronal viability with increase in length of neurite outgrowth (31% for LPS + ASH-WEX and 52% for LPS + FIV) and reduced the percentage of apoptotic neurons up to 15% (ASH-WEX) and 11% (FIV) as compared to LPS alone-treated co-cultures (Fig. 7b-d; *p* ≤ 0.05).

## Discussion

Peripheral inflammatory responses elicited by single i.p. dose of LPS were observed to result in several behavioral deficits in the animals. Diminished social and environmental exploration, poor learning and memory and impaired neuromuscular coordination etc. were observed in these animals which are collectively termed as LPS-induced sickness behavior [47]. During locomotor coordination test, LPS-treated rats were lethargic as discerned by maximum number of falls and less amount of time spent on the rotating rod, whereas, animals orally fed with ASH-WEX before LPS treatment performed well on the rotating rod with less number of falls indicating normal neuromuscular coordination (Fig. 1a-b). Similarly in Narrow beam walking test, LPS-treated rats took longer time to cross the beam with poor balance and higher frequency of paw slippages while ASH-WEX supplemented rats transverse the beam in shorter span of time like control rats (Fig. 1c-d). Further, during NOR test, LPS-treated animals preferred old object over the novel one (PI ≤ 0.5) with more frequency of episodes and time spent in exploring old object, whereas, ASH-WEX pretreated group highly preferred the novel object (PI ≥ 0.5) with more time spent in exploration of novel object (Fig. 2a-c). These observations from NOR test suggest ASH-WEX improved memory and learning which got impaired due to sickness behavior associated with LPS-induced inflammatory responses.

Healthy animals exhibit spontaneous self paced grooming defined by a stereotyped cephalocaudal sequence [48, 49]. Conversely animals with high stress and anxiety levels show interrupted and disorganized grooming pattern. LPS injected rats showed anxious behavior along with less number and duration of grooming bouts with disrupted and disorganized pattern which may be attributed to sickness behavior induced by LPS. This finding was consistent with other studies reporting that i.p. injection of LPS caused the reduction in grooming bouts and fur cleaning numeric score in inbred mice [50]. ASH-WEX supplementation attenuated anxiety-like behavior and improved frequency and duration of grooming bouts with organized and structured pattern in these animals, suggesting anxiolytic activity of Ashwagandha (Fig. 2d-e).

These observations were also supported by changes in rearing activity which is an indices of exploratory behavior found in rodents [51]. LPS + ASH-WEX- and ASH-WEX alone-treated rats showed maximum number of rearing episodes compared to LPS-treated rats indicative of improved exploratory behavior (Fig. 2f). Various experimental and clinical studies have reported memory enhancing and learning improvement potential of alcoholic and water based formulation of Ashwagandha [52]. Recently our lab reported that aqueous extract of Ashwagandha has immunomodulatory and anxiolytic effects and improved memory retention and learning ability by alleviating cellular stress via regulating synaptic plasticity and cell survival signaling cascades in acute sleep deprived and HFD fed obese rats [32, 33, 35, 53].

Different brain regions have differential susceptibility to LPS-induced neuroinflammation with hippocampus and pyriform cortex (PC) being highly affected areas. Since hippocampus region is well known for its role in synaptic plasticity, regulation of spatial and learning memory, so expression of synaptic plasticity and neuronal survival protein markers were studied in hippocampus and PC regions. Further detailed study of molecular mechanisms revealed that LPS significantly upregulated the expression of 140 and 180 kDa isoforms of NCAM along with PSA-NCAM in hippocampus and PC regions. However, ASH-WEX pretreatment normalized the inflammation induced increase in PSA-NCAM and NCAM expression and seems to play important role in facilitating memory consolidation and regulating synaptic plasticity (Fig. 3). Both PSA-NCAM and NCAM expression is reported to be upregulated under various stress conditions and mood disorders [54, 55]. Enhanced expression of both NCAM and PSA-NCAM in LPS-treated rats may be the compensatory neuroprotective mechanism in stress vulnerable neuronal circuits to combat stressful conditions. They may be instrumental to maintain neural networking since repair and functional recovery after brain injury critically depends on structural and functional plasticity of preserved neuronal networks. Menzel et al. (2016) reported that antibody blocking of neural cell adhesion molecule L1 inhibits activated immune T cell adhesion and attack to neurons in co-cultures thereby reduces the neurodegeneration [56]. The study suggested that downregulation of L1 is an adaptive endeavor to promote neuronal self defense in response to neuroinflammation and mitigates inflammatory neuronal injury.

LPS administration to animals downregulated CaMKIIα protein expression in hippocampus region, while its expression remained unaffected in PC region of brain. Interestingly ASH-WEX supplementation to LPS-treated rats enhanced CaMKIIα protein expression. On the other hand, expression of calcineurin was found to be unaffected in all the groups in both regions of brain (Fig. 3). Functionally, calcium and calmodulin serine/ threonine protein kinases CaMKII and protein phosphatases calcineurin coordinately regulate the calcium dependent synaptic plasticity including LTP. They also play key role in release of neurotransmitters, learning and memory consolidation [57]. Decrease in expression of CaMKIIα in LPS-treated rats may be associated with performance deficits as observed in NOR test. Zhang et al. (2015) also reported that inflammatory trigger Aβ, elevated the Ca^2+^ levels and reduced CaMKII activity leading to degeneration of APPKI (amyloid precursor protein knock in) neurons along with impairment of calcium dependent synaptic plasticity and memory loss in AD [58]. ASH-WEX pretreated group showed attenuation of cognitive deficits, promoted structural and functional integrity as well as improved working memory and learning functions thus suggesting their potential role against inflammation mediated neurodegeneration.

LPS treatment also significantly decreased the expression of neuronal markers such as GAP43, MAP2 which are responsible for maintenance of neuronal structure and function. LPS + ASH-WEX- and ASH-WEX alone-treated rats exhibited near control level expression of MAP2 and GAP43 (Fig. 3) which may mediate protection of neurons by ASH-WEX from neuroinflammation associated degeneration as observed in conditioned medium-treated neuronal cultures and microglial-neuronal co-cultures (Fig. 5). Various previous studies have also reported that alcoholic extract of Ashwagandha leaves and roots promote neuronal survival and neurite outgrowth via upregulated expression of BDNF, ARC, NF200, MAP2, PSD95, GAP43 [19, 59, 60].

Accumulating evidence support that BDNF-TrkB is a major pathway implicated in the plethora of brain functions including neuronal cell survival, neurite growth, cell migration, synapse formation etc. [61, 62]. LPS treatment lead to reduction in mRNA expression of BDNF and its TrkB receptor as well asTrkB receptor protein expression in the hippocampus and PC regions suggesting that the deterioration of synaptic plasticity in LPS-treated rats may be mediated by dysfunctional BDNF-TrkB pathway (Fig. 4). Various previous studies have also reported that administration of LPS leads to significant reduction of BDNF mRNA and protein expression in hippocampus and different cortical regions resulting in depressive like sickness behavior and cognitive impairments in these animals [63, 64]. Interestingly, ASH-WEX pretreatment suppressed these LPS-induced changes in BDNF and TrkB receptor mRNA as well as protein expression thus indicating the potential activity of ASH-WEX in maintaining neuronal plasticity and recovery of functional deficits, working memory and learning.

BDNF binding causes tyrosine phosphorylation in the intracellular Trk domains which in turn triggers PLCγ-IP3 and PI3K-Akt intracellular cascades thereby initiating the transduction of BDNF signal [61, 65]. Downregulation in mRNA expression of PI3K and Akt and further phosphorylated form of Akt in LPS-treated rats may suggest suppression of cell survival signals which was restored with ASH-WEX supplementation. Phosphorylated Akt then promotes cell survival and inhibits apoptosis through inactivation of apoptotic protein BAD with corresponding activation of Bcl-xL thus protecting the cell from apoptotic stimuli. LPS-induced decrease in expression of Bcl-xL was also restored by ASH-WEX pretreatment thus indicating anti-apoptotic potential of ASH-WEX against inflammation associated neurotoxicity (Fig. 4). Parallel study using in vitro culture system clearly showed induction of neurotoxicity in neuronal cultures exposed to LPS stimulated microglial conditioned medium and microglial-neuronal co-cultures which may be explained by in vivo observation of suppression of PI3K-Akt signaling by LPS. ASH-WEX and FIV pretreatment to LPS-treated cultures may have maintained the neuronal health by regulating cell survival PI3K-Akt pathway. Hwang et al. (2017) also reported that aqueous methanolic extract of Ashwagandha roots and its active ingredients withaferin A, withanolide A protect the hippocampal neurons from nutritional stress by upregulating the expression of BDNF and PI3K-Akt baseline levels [66]. Likewise in BDNF triggered PLCγ signaling cascade, ASH-WEX supplementation also restored PLCγ and IP3R mRNA expression which was downregulated by LPS (Fig. 4).

Further, we observed that conditioned media-treated neuronal cultures (CM-LPS) showed reduced MAP2 immunoreactivity while ASH-WEX and FIV treatment normalized its expression (Fig. 5). These changes were also accompanied by significant increase in the number of apoptotic neurons with disintegrated and condensed nuclei as revealed by DAPI staining (Fig. 6). The extent of MAP2 loss is directly attributed to neuronal death and altered expression of MAP2 in remaining neurons may impact the dynamics of dendritic structures [67]. Recent studies have reported prominent microgliosis overlapped with decreased MAP2 immunoreactivity and phosphorylation in CA1 stratum radiatum area at 2 and 3 weeks after status epilepticus [68, 69]. CM-LPS-treated neuronal cultures with ASH-WEX and FIV supplementation showed high percentage of the viable cells and restoration of neurite outgrowth. A recent report by Saykally et al. (2017) also showed that treatment of cultured traumatic brain injury (TBI) model neurons with *Withania somnifera* root chloroform-methanolic extract protected neurons from TBI injury induced apoptotic cell death along with restoration of neurite length and number [70]. Various active phytochemicals like withaferin A, withanone, withanolide A, withanoside IV and withanoside VI etc. present in Ashwagandha are found to be involved in its neuroprotective activity [17, 59, 71–73]. Array of secreted factors from activated glia like NO, NO derivatives and cytokines either lead to neuronal death or interfere with cytoskeleton reorganization of neurons which is also implicated in normal functioning of synaptic networks [74, 75]. We have earlier reported that ASH-WEX and FIV contain withaferin A and withanone as active components which were shown to inhibit the production of various inflammatory mediators like ROS, NO, TNF-α, IL-1β, IL-6 etc. in LPS activated primary microglial cultures as well as murine BV-2 cells [27].

ASH-WEX and FIV also restored the loss of neuronal structure and function in extracts pretreated activated microglia/ neuronal co-cultures (Fig. 7). Microglial and neuronal interactions involve array of ligands and receptors influencing the normal homeostasis in the brain during both physiological and inflammatory conditions [76]. Neuronal CD200–microglial CD200R interaction was reported to play critical role in determining the neuronal protection in setting of inflammation–mediated neurodegeneration, where this neuronal-microglial crosstalk gets altered. The disturbed equilibrium results in disturbance of immunoregulatory system contributing to microglial activation, chronic neuroinflammation and neuronal damage in various neurological disorders [77, 78]. Our current data suggests that both altered surface receptor molecules and continuous release of inflammatory mediators from activated microglia present in co-cultures may be collectively responsible for LPS-induced neuroinflammation and subsequent neurodegeneration.

## Conclusion

**Fig. 8:**
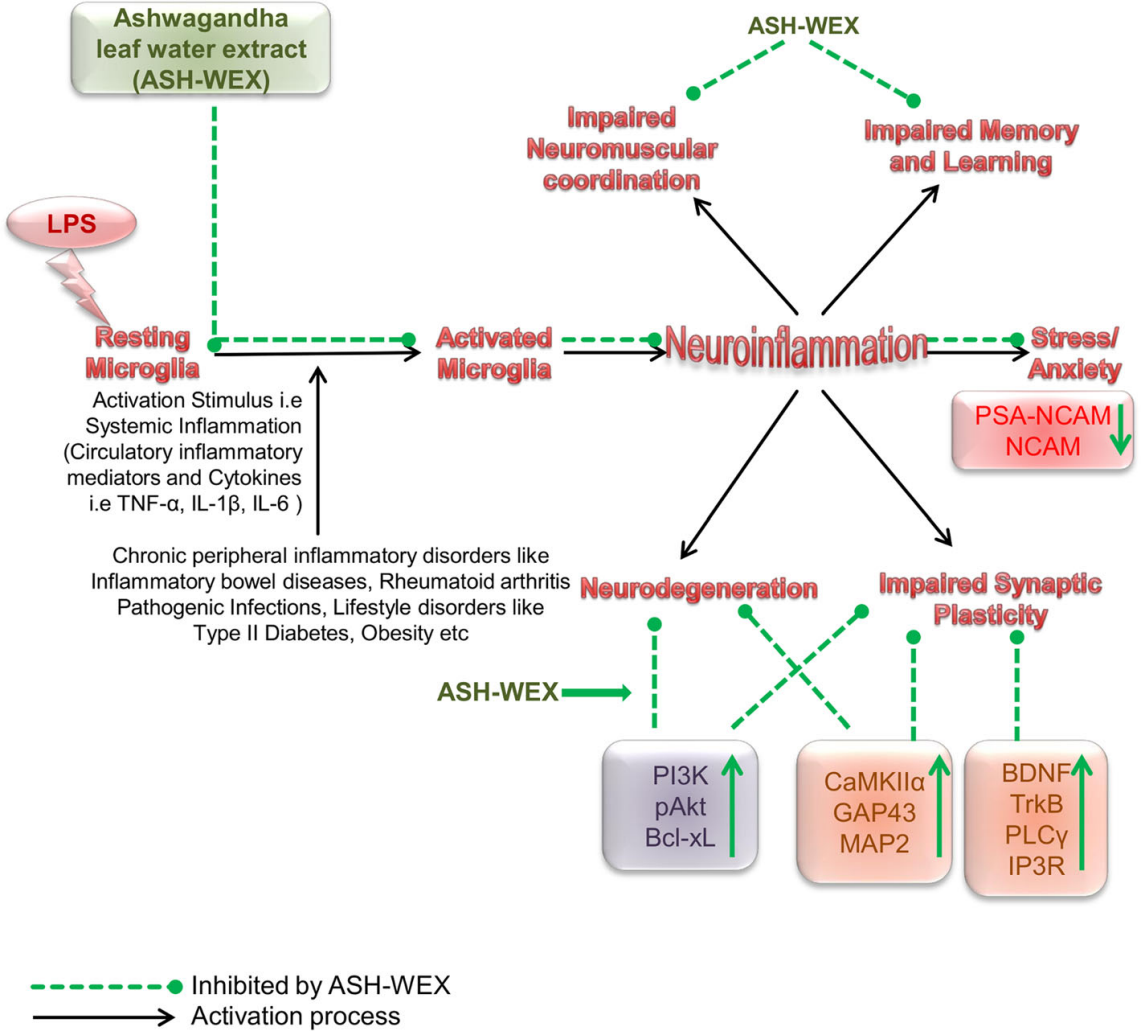
Graphical representation of the study LPS (i.p.) induced sickness behavior, systemic inflammation driven neuroinflammation, and ultimately leads to neurodegeneration by adversely affecting key pathways involved in synaptic plasticity and cell survival. ASH-WEX supplementation reduced stress, sickness behavior and promoted the working memory and learning functions as well as neuro-muscular coordination impaired by LPS treatment. Ashwagandha exhibits its pleotropic neuroprotective activity by targeting various pathways and proteins involved in synaptic plasticity and cell survival such as BDNF-TrkB, PLCγ-IP3, PI3K-Akt pathways thereby promoting neuronal health and vitality. (↑ indicates “upregulation”, ↓ indicates “downregulation”, dotted arrows in green color indicate “inhibitory effects mediated by ASH-WEX”, and solid black arrows indicate “activation”)

This is the first comprehensive study wherein an attempt has been made to link systemic inflammation with CNS inflammation and neurodegeneration simultaneously using in vitro and in vivo model systems. The current data provides scientific evidence that oral feeding of leaf water extract of Ashwagandha prevented the LPS-induced neuroinflammation, neurodegeneration, cognitive decline, impairments in synaptic plasticity and improved working memory and learning and locomotor coordination. The signaling pathways involved may be BDNF-TrkB, PLCγ-IP3 and PI3K-Akt which are already known to regulate neuronal survival and maintenance of various other synaptic plasticity proteins (Fig. 8). ASH-WEX and FIV were also found to inhibit apoptosis of neurons from the inflammatory mediators secreted by inflamed microglia as well as due to altered microglial – neuronal crosstalk crucial for neuronal functioning. The current data suggests that water extract from *Withania somnifera* leaves may be a potential candidate to attenuate neuroinflammation and neurodegeneration induced by systemic inflammation. Moreover simultaneous focus on ‘behavioral’ and ‘molecular’ mechanisms that elucidated the potential beneficial effects of ASH-WEX in this preclinical study may significantly contribute to plan future translational research on *Withania somnifera* (also known as “Queen of Ayurveda” due to its vast medicinal properties) as a source of natural medicines for neuroprotection and to treat neuroinflammation associated with NDDs.

## Abbreviations

ASH-WEX: Ashwagandha leaf water extract
BAD: BCL2 associated death promoter
BCL2: B-cell lymphoma 2
Bcl-xL: B-cell lymphoma 2 extra large
BDNF: Brain derived neurotrophic factor
CaN: Calcineurin
CM: Conditioned Medium
DAPI: 4′,6-diamidino-2-phenylindole
FIV: Active chloroform fraction (Fraction IV)
GAP43: Growth associated protein 43
GAPDH: Glyceraldehyde 3-phosphate dehydrogenase
HFD: High Fat Diet
i.p: Intraperitoneal injection
Iba-1: Ionized calcium-binding Adaptor Molecule 1
IDV: Integrated density values
IL-1β: Interleukin beta
IL-6: Interleukin 6
iNOS: Inducible nitric oxide synthase
IP3: inositol 1,4,5- triphosphate
IκB: IkappaB kinase
LPS: Lipopolysaccharide
MAP2: Microtubule–associated protein 2
MAPK: Mitogen activated protein kinase
NCAM: Neural Cell adhesion molecule
NFκB: Nuclear factor kappa light-chain-enhancer of activated B cells
NMDA: N-methyl-D-aspartate
NOR Test: Novel Object Recognition Test
PC: Pyriform Cortex
PI score: Preference index score
PI: Propidium iodide
PI3K: Phosphoinositide 3-kinase
PLCγ: Phosphoinositide phospholipase C gamma
PSA-NCAM: Polysialylated-neural cell adhesion molecule
PSD95: Post synaptic density protein 95
RNS: Reactive nitrogen species
ROS: Reactive oxygen species
TNF-α: Tumor necrosis factor alpha
TrkB: Tropomyosin receptor kinase B
